# Healthcare Costs of Metastatic Cutaneous Melanoma in the Era of Immunotherapeutic and Targeted Drugs

**DOI:** 10.3390/cancers12041003

**Published:** 2020-04-18

**Authors:** Brenda Leeneman, Carin A. Uyl-de Groot, Maureen J. B. Aarts, Alexander C. J. van Akkooi, Franchette W. P. J. van den Berkmortel, Alfons J. M. van den Eertwegh, Jan Willem B. de Groot, Karin H. Herbschleb, Jacobus J. M. van der Hoeven, Geke A. P. Hospers, Ellen Kapiteijn, Djura Piersma, Rozemarijn S. van Rijn, Karijn P. M. Suijkerbuijk, Albert J. ten Tije, Astrid A. M. van der Veldt, Gerard Vreugdenhil, Michel W. J. M. Wouters, John B. A. G. Haanen, Margreet G. Franken

**Affiliations:** 1 Department of Health Technology Assessment, Erasmus School of Health Policy & Management, Erasmus University Rotterdam, Burgemeester Oudlaan 50, 3062 PA Rotterdam, The Netherlands; uyl@eshpm.eur.nl (C.A.U.-d.G.); franken@imta.eur.nl (M.G.F.); 2 Institute for Medical Technology Assessment, Erasmus University Rotterdam, Burgemeester Oudlaan 50, 3062 PA Rotterdam, The Netherlands; 3 Department of Medical Oncology, Maastricht University Medical Center+, P. Debyelaan 25, 6229 HX Maastricht, The Netherlands; mjb.essers.aarts@mumc.nl; 4 Department of Surgical Oncology, Netherlands Cancer Institute, Antoni van Leeuwenhoek, Plesmanlaan 121, 1066 CX Amsterdam, The Netherlands; a.v.akkooi@nki.nl (A.C.J.v.A.); m.wouters@nki.nl (M.W.J.M.W.); 5 Department of Medical Oncology, Zuyderland Medical Center, Dr. H. van der Hoffplein 1, 6162 BG Sittard-Geleen, The Netherlands; f.vandenberkmortel@zuyderland.nl; 6 Department of Medical Oncology, Cancer Center Amsterdam, Amsterdam UMC, Vrije Universiteit Amsterdam, De Boelelaan 1118, 1182 DB Amsterdam, The Netherlands; vandeneertwegh@vumc.nl; 7 Oncology Center Isala, Isala, Dokter van Heesweg 2, 8025 AB Zwolle, The Netherlands; j.w.b.de.groot@isala.nl; 8 Department of Medical Oncology, Radboud University Medical Center, Geert Grooteplein Zuid 10, 6525 GA Nijmegen, The Netherlands; k.herbschleb@antoniusziekenhuis.nl (K.H.H.); koos.vanderhoeven@radboudumc.nl (J.J.M.v.d.H.); 9 Department of Medical Oncology, University Medical Center Groningen, University of Groningen, Hanzeplein 1, 9713 GZ Groningen, The Netherlands; g.a.p.hospers@umcg.nl; 10 Department of Medical Oncology, Leiden University Medical Center, Albinusdreef 2, 2333 ZA Leiden, The Netherlands; h.w.kapiteijn@lumc.nl; 11 Department of Internal Medicine, Medisch Spectrum Twente, Koningsplein 1, 7512 KZ Enschede, The Netherlands; d.piersma@mst.nl; 12 Department of Internal Medicine, Medical Center Leeuwarden, Henri Dunantweg 2, 8934 AD Leeuwarden, The Netherlands; rozemarijn.van.rijn@znb.nl; 13 Department of Medical Oncology, UMC Utrecht Cancer Center, Heidelberglaan 100, 3584 CX Utrecht, The Netherlands; k.suijkerbuijk@umcutrecht.nl; 14 Department of Internal Medicine, Amphia Hospital, Molengracht 21, 4818 CK Breda, The Netherlands; atentije@amphia.nl; 15 Department of Medical Oncology, Erasmus MC Cancer Institute, Doctor Molewaterplein 40, 3015 GD Rotterdam, The Netherlands; a.vanderveldt@erasmusmc.nl; 16 Department of Internal Medicine, Maxima MC, Dominee Theodor Fliednerstraat 1, 5631 BM Eindhoven, The Netherlands; g.vreugdenhil@mmc.nl; 17 Scientific Bureau, Dutch Institute for Clinical Auditing, Rijnsburgerweg 10, 2333 AA Leiden, The Netherlands; 18 Department of Medical Oncology, Netherlands Cancer Institute, Antoni van Leeuwenhoek, Plesmanlaan 121, 1066 CX Amsterdam, The Netherlands; j.haanen@nki.nl

**Keywords:** metastatic melanoma, healthcare costs, real-world data, immunotherapy, targeted therapy

## Abstract

Immunotherapeutic and targeted drugs improved survival of patients with metastatic melanoma. There is, however, a lack of evidence regarding their healthcare costs in clinical practice. The aim of our study was to provide insight into real-world healthcare costs of patients with metastatic cutaneous melanoma. Data were obtained from the Dutch Melanoma Treatment Registry for patients who were registered between July 2012 and December 2018. Mean total/monthly costs per patient were reported for all patients, patients who did not receive systemic therapy, and patients who received systemic therapy. Furthermore, mean episode/monthly costs per line of therapy and drug were reported for patients who received systemic therapy. Mean total/monthly costs were € 89,240/€ 6809: € 7988/€ 2483 for patients who did not receive systemic therapy (*n* = 784) and € 105,078/€ 7652 for patients who received systemic therapy (*n* = 4022). Mean episode/monthly costs were the highest for nivolumab plus ipilimumab (€ 79,675/€ 16,976), ipilimumab monotherapy (€ 79,110/€ 17,252), and dabrafenib plus trametinib (€ 77,053/€ 12,015). Dacarbazine yielded the lowest mean episode/monthly costs (€ 6564/€ 2027). Our study showed that immunotherapeutic and targeted drugs had a large impact on real-world healthcare costs. As new drugs continue entering the treatment landscape for (metastatic) melanoma, it remains crucial to monitor whether the benefits of these drugs outweigh their costs.

## 1. Introduction

The global incidence of cutaneous melanoma has been increasing over the past decades [1]. In The Netherlands, the estimated incidence rate increased from 8.2 to 24.2 per 100,000 person-years between 1990 and 2018. Most patients (approximately 85%) are diagnosed with localized melanoma and have a relatively good prognosis. Melanoma has, however, a strong tendency to metastasize, resulting in a poor prognosis. Historically, one- and five-year survival rates of patients with metastatic melanoma were only 39% and 12%, respectively [2].

Until 2011, treatment options for metastatic melanoma were limited to chemotherapy (including dacarbazine and temozolomide) and interleukin-2. However, these drugs never demonstrated to improve survival [3,4,5]. Advances in the development of immunotherapeutic and targeted drugs dramatically changed the treatment landscape. In 2011, the first two new drugs were approved by the U.S. Food and Drug Administration: ipilimumab (an anti-CTLA-4 antibody) and vemurafenib (a BRAF inhibitor) [6]. European approval by the European Medicines Agency followed in the same year for ipilimumab and in 2012 for vemurafenib [7]. Since then, several other drugs and combinations of drugs have been approved for the treatment of metastatic melanoma (Table S1) [6,7].

Although the new drugs demonstrated to improve survival [8], there is a lack of evidence regarding their healthcare costs in real-world clinical practice. Previous studies only reported real-world healthcare costs of ipilimumab and vemurafenib [9,10,11]. Therefore, the aim of our study was to provide insight into real-world healthcare costs of patients with metastatic cutaneous melanoma in The Netherlands since the approval of the new immunotherapeutic and targeted drugs.

## 2. Results

### 2.1. Baseline Patient and Tumor Characteristics

A total of 4806 patients were included in our study. The median age was 64 years; 59% of the patients were male (Table 1). Most patients had a good Eastern Cooperative Oncology Group (ECOG) performance status (i.e., 0 or 1; 74%), a normal lactate dehydrogenase (LDH) level (58%), and were diagnosed with M1c disease (69%). More than one-third of the patients with M1c disease had brain metastases (39%).

Of all patients, 16% (*n* = 784) did not receive systemic therapy during the study period and 84% (*n* = 4022) received at least one systemic therapy. Patients who received systemic therapy had more favorable baseline patient and tumor characteristics than patients who did not receive systemic therapy. They were younger (median age: 63 versus 72 years), had more often a good ECOG performance status (80% versus 44%) and a normal LDH level (60% versus 46%), and had less often brain metastases (36% versus 58% of the patients with M1c disease).

### 2.2. Healthcare Costs of All Patients

Table 2 presents the healthcare resource use and costs of all patients (*n* = 4806). The mean (median) observation period was 18.0 (12.1) months; 66% of the patients died during this period. Mean total costs were € 89,240 (standard deviation (SD): € 86,489). Systemic therapy was by far the most important cost driver, accounting for 83% of the costs (€ 73,998). On average, patients received 1.4 lines of therapy. The remaining 17% of the costs was related to hospital admissions (6%; € 5363), hospital visits (5%; € 4287), medical imaging (2%; € 2086), radiotherapy (1%; € 1318), surgery (1%; € 1224), genetic testing (1%; € 891), hyperthermia (<1%; € 70), and radiofrequency ablation (RFA; <1%; € 2). Mean monthly costs were € 6809 (SD: € 5783).

### 2.3. Healthcare Costs of Patients Who Did not Receive Systemic Therapy

The mean (median) observation period of patients who did not receive systemic therapy (*n* = 784) was 11.7 (3.7) months (Table 2). Mean total costs were € 7988 (SD: € 7490). These costs were mainly driven by the costs of hospital admissions, which accounted for 35% of the costs (€ 2831). Almost half of all admissions (44%) was related to palliative care. The remaining 65% of the costs was attributable to surgery (15%; € 1160), medical imaging (14%; € 1080), radiotherapy (13%; € 1068), hospital visits (13%; € 1010), genetic testing (9%; € 753), hyperthermia (1%; € 83), and RFA (<1%; € 2). Mean monthly costs were € 2483 (SD: € 3191).

Of the patients who did not receive systemic therapy, 81% (*n* = 634) died during the observation period and 19% (*n* = 150) was still alive at the cutoff date. Their baseline patient and tumor characteristics are presented in Table S2. Deceased patients had less favorable baseline characteristics than patients who were still alive. They were older (median age: 73 versus 65 years), had less often a good ECOG performance status (41% versus 58%) and a normal LDH level (43% versus 60%), and were more often diagnosed with M1c disease (71% versus 27%). Table 3 presents the healthcare costs of these patients. Mean total costs were lower for deceased patients than for patients who were still alive (€ 7219 versus € 11,237). Their mean monthly costs were, however, much higher (€ 2981 versus € 378). Costs of deceased patients were mainly driven by the costs of hospital admissions (41%; € 2961). Surgery (27%; € 3039) and medical imaging (21%; € 2350) were the main cost drivers for patients who were still alive.

### 2.4. Healthcare Costs of Patients Who Received Systemic Therapy

The mean (median) observation period of patients who received systemic therapy (*n* = 4022) was 19.3 (13.5) months; approximately two-thirds of the patients (63%) died during this period (Table 2). Mean total costs were € 105,078 (SD: € 85,963). Systemic therapy was the main cost driver (84%; € 88,422), followed by hospital admissions (6%; € 5857), hospital visits (5%; € 4926), medical imaging (2%; € 2282), radiotherapy (1%; € 1367), surgery (1%; € 1236), genetic testing (1%; € 918), hyperthermia (<1%: € 68), and RFA (<1%; € 3). Mean monthly costs were € 7652 (SD: € 5798).

Table 4 presents the episode and monthly costs stratified by line of therapy. In total, 2107 patients received one line of therapy, 1077 patients received two lines of therapy, and 838 patients received three (or more) lines of therapy. Pembrolizumab was the most frequently prescribed drug in the first line (21%), ipilimumab in the second line (23%), and dabrafenib plus trametinib in the third line (28%). Mean episode costs were the highest for the second line (€ 59,701) and the lowest for the third line (€ 49,725). The mean monthly costs were also the highest for the second line (€ 11,939), but the lowest for the first line (€ 8231).

**Figure 1:**
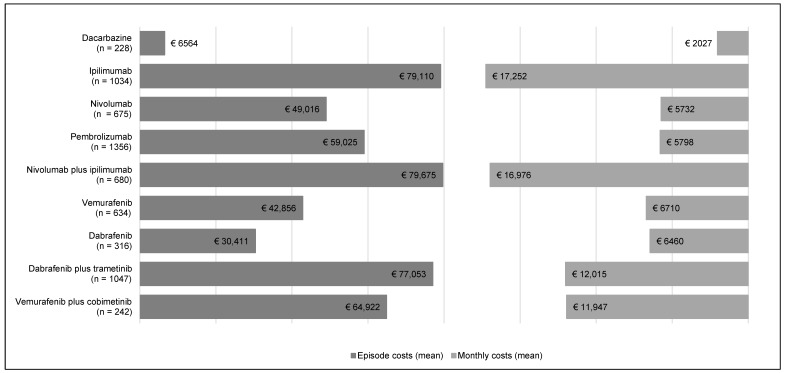
Episode and monthly costs stratified by drug.

Figure 1 presents the episode and monthly costs stratified by drug. Mean episode costs were the highest for nivolumab plus ipilimumab (€ 79,675; SD: € 44,196), followed by ipilimumab monotherapy (€ 79,110; SD: € 29,113) and dabrafenib plus trametinib (€ 77,053; SD: € 63,451). Dacarbazine yielded the lowest mean episode costs (€ 6564; SD: € 5090). The mean monthly costs were also the highest for nivolumab plus ipilimumab and ipilimumab monotherapy (€ 16,976 and € 17,252, respectively) and the lowest for dacarbazine (€ 2027). Mean monthly costs were similar between drugs within the same class: vemurafenib and dabrafenib (€ 6710 and € 6460, respectively), dabrafenib plus trametinib and vemurafenib plus cobimetinib (€ 12,015 and € 11,947, respectively), and nivolumab and pembrolizumab (€ 5732 and € 5798, respectively). Detailed results regarding the episode costs stratified by drug are presented in Table S3.

## 3. Discussion

This study provides insight into real-world healthcare costs of patients with metastatic cutaneous melanoma in The Netherlands since the approval of the new immunotherapeutic and targeted drugs. Mean total costs were € 89,240 (SD: € 86,489). Costs substantially differed between patients who did not receive systemic therapy (€ 7988) and patients who received systemic therapy (€ 105,078). This difference was largely owing to the costs of systemic therapy, which accounted for more than 80% of the costs.

Patients who did not receive systemic therapy were stratified by vital status because we assumed that these patients either had an infaust prognosis or a rather good prognosis (e.g., patients with oligometastatic disease). The results of our study confirm this assumption. First, deceased patients had less favorable baseline patient and tumor characteristics than patients who were still alive (Table S2). Second, the observation period was much shorter for deceased patients than for patients who were still alive (mean: 5.4 versus 38.2 months). Finally, hospital admissions were the main cost driver for deceased patients (41%), whereas costs of patients who were still alive were mainly driven by the costs of surgery (27%) and medical imaging (21%).

For patients who received systemic therapy, costs were stratified by drug. Although episode costs differed between drugs within the same class (vemurafenib and dabrafenib, dabrafenib plus trametinib and vemurafenib plus cobimetinib, and nivolumab and pembrolizumab), their monthly costs were similar. This underlines the importance of accounting for differences in episode durations (and observation periods). Moreover, a network meta-analysis (NMA) showed that effectiveness and safety were also comparable between drugs within the same class [12]. Therefore, it could be suggested that clinicians should not be restricted by differences in effectiveness, safety, and costs while choosing between these drugs.

Furthermore, our study showed that episode costs were similar between ipilimumab monotherapy and nivolumab plus ipilimumab. This was mainly owing to the costs of ipilimumab, which were higher for ipilimumab monotherapy (€ 70,976) than for ipilimumab in combination with nivolumab (€ 55,228). On average, patients received 3.2 cycles of ipilimumab monotherapy compared to 2.6 cycles of ipilimumab combination therapy. Due to a reasonably comparable episode duration (mean: 9.1 versus 9.6 months), monthly costs were also similar between ipilimumab monotherapy and nivolumab plus ipilimumab. The previously mentioned NMA showed, however, that effectiveness was in favor of nivolumab plus ipilimumab, whereas safety was in favor of ipilimumab monotherapy [12]. This underlines that evidence on effects, costs, and cost-effectiveness is crucial. It will provide insight into what extends the benefits of drugs outweighing their costs, which may facilitate evidence-based decision-making in clinical practice.

Three previous studies reported real-world healthcare costs of ipilimumab and vemurafenib. One of these studies was our own study in which we calculated healthcare costs of all Dutch patients who received ipilimumab [11]. The two other studies calculated healthcare costs of United States (US) patients who received ipilimumab or vemurafenib. According to the study by Chang et al. [9], mean episode costs were US$ 153,062 (≈€ 113,480) for ipilimumab and US$ 77,687 (≈€ 57,597) for vemurafenib. In the study by Toy et al. [10], mean monthly costs were US$ 35,472 (≈€ 26,718) for ipilimumab and US$ 17,793 (≈€ 13,402) for vemurafenib. Both of these studies reported considerably higher costs than our study. It is, however, difficult to compare costs between countries as, for example, drug use and unit prices may differ. This information was not reported in both studies.

It should be noted that our study has some limitations. First, we used list prices for drugs, and reference prices and tariffs for other resources. Although these prices may not reflect actual costs (e.g., nivolumab and pembrolizumab are subjected to a confidential financial arrangement), the use of these sources is recommended in the Dutch costing manual [13]. Second, we did not include healthcare costs outside the hospital setting, such as costs of hospice care, which may have led to an underestimation of the actual healthcare costs. We believe, however, that the impact will be rather limited because costs were mainly driven by the costs of systemic therapy. Third, approximately 10% of the patients received at least one investigational drug. Costs of these drugs are paid by pharmaceutical companies. However, in our study, costs of investigational drugs were only set at zero if the drug was given in a blinded trial or if the drug was not approved for metastatic melanoma in The Netherlands at the time of this study. If costs of all investigational drugs were set at zero, mean total costs of patients who received systemic therapy would have been € 102,450 instead of € 105,078. Finally, costs were not yet complete for all patients because 34% of the patients were still alive at the cutoff date. These patients will accrue additional costs during the remainder of their life.

## 4. Materials and Methods

### 4.1. Data Source and Patient Population

Data were obtained from the population-based Dutch Melanoma Treatment Registry (DMTR). The DMTR contains detailed data regarding baseline patient and tumor characteristics, treatment patterns, healthcare resource use, and survival of all Dutch patients with unresectable stage IIIC or stage IV melanoma (i.e., metastatic melanoma). In compliance with Dutch regulations, the DMTR was approved by the medical ethical committee and was not subject to the Medical Research Involving Human Subjects Act. A detailed description of the DMTR has been previously published [14].

For this study, we selected all patients (≥18 years) with metastatic cutaneous melanoma who were registered in the DMTR between July 2012 and December 2018. Patients with incomplete data regarding the start or stop date of a systemic therapy and/or patients with insufficient follow-up (i.e., patients who were alive at the cutoff date with an observation period of less than six months) were excluded. The cutoff date was December 2019.

### 4.2. Cost Analysis

The cost analysis was conducted from a hospital perspective using the methodology as described in the Dutch costing manual [13]. Costs were calculated by applying unit costs to individual patient resource use for the following cost components: medical imaging, genetic testing, hospital visits, hospital admissions, surgery, radiotherapy, hyperthermia, RFA, and systemic therapy. Missing data on resource use were imputed using conditional mean imputation. Table 5 presents the unit costs. Unit costs of medical imaging, genetic testing, surgery, radiotherapy, hyperthermia, and RFA were based on tariffs issued by the Dutch Healthcare Authority [15]. The unit costs of hospital visits and hospital admissions were derived from the Dutch costing manual [13]. Drug costs were acquired from the Z-index (i.e., the Dutch drug database) for two chemotherapeutic drugs (dacarbazine and temozolomide), three immunotherapeutic drugs (ipilimumab, nivolumab, and pembrolizumab), and six targeted drugs (vemurafenib, dabrafenib, trametinib, cobimetinib, encorafenib, and binimetinib) [16]. Costs of investigational drugs were set at zero if the drug was given in a blinded trial or if the drug was not approved for metastatic melanoma in The Netherlands at the time of this study. All costs were based on Euro 2018 cost data. Where necessary, costs were adjusted to 2018 prices using the consumer price index from Statistics Netherlands [17].

### 4.3. Data Analysis

Baseline patient and tumor characteristics were summarized using descriptive statistics. Age was presented as mean and SD as well as median and interquartile range. Gender, ECOG performance status, LDH level, M category (i.e., site of distant metastases according to the seventh edition of the American Joint Committee on Cancer staging manual), and brain metastases were presented as counts and proportions.

Costs were reported for all patients irrespective of their treatment status. To provide further details, costs were also separately reported for patients who did not receive systemic therapy during the study period stratified by vital status (dead or alive) and patients who received at least one systemic therapy stratified by line of therapy and drug. Due to low numbers of patients, costs were only separately reported for the first, second, and third lines. Similarly, costs were not separately reported for temozolomide and encorafenib plus binimetinib. Mean (SD) total costs per patient were calculated from the diagnosis of metastatic melanoma until death or last follow-up (i.e., the observation period). Mean (SD) episode costs per line of therapy and drug were calculated from the diagnosis of metastatic melanoma or the start of a systemic therapy until the start of a new systemic therapy, death, or last follow-up (i.e., the episode duration). To account for differences in observation periods or episode durations, costs were also reported as mean (SD) monthly costs. These costs were calculated by dividing the total costs by the observation period and the episode costs by the episode duration. All analyses were conducted using STATA statistical analysis software, version 16.0 (StataCorp. 2019. Stata Statistical Software: Release 16. College Station, TX: StataCorp LLC, Texas, USA).

## 5. Conclusions

Our study showed that immunotherapeutic and targeted drugs had a large impact on real-world healthcare costs of patients with metastatic melanoma. Compared to dacarbazine, episode costs were five times higher for dabrafenib and 12 times higher for nivolumab plus ipilimumab, ipilimumab monotherapy, and dabrafenib plus trametinib. As new drugs continue entering the treatment landscape for (metastatic) melanoma, it remains crucial to monitor whether the benefits of these drugs outweigh their costs.

## Figures and Tables

**Table 1:** Baseline patient and tumor characteristics.

	**All Patients**	**Patients Who Did Not Receive Systemic Therapy**	**Patients Who Received Systemic Therapy**
	***n* = 4806**	***n* = 784**	***n* = 4022**
**Age, *years***			
Mean (SD)	63 (13)	70 (13)	62 (13)
Median (IQR)	64 (54–73)	72 (62–80)	63 (53–71)
**Gender, *n* (%)**			
Male	2813 (59%)	447 (57%)	2366 (59%)
Female	1992 (41%)	336 (43%)	1656 (41%)
Unknown	1 (0%)	1 (0%)	0 (0%)
**ECOG performance status, *n* (%)**			
0	2168 (45%)	155 (20%)	2013 (50%)
1	1407 (29%)	193 (25%)	1214 (30%)
≥2	623 (13%)	209 (27%)	414 (10%)
Unknown	608 (13%)	227 (29%)	381 (9%)
**LDH level, *n* (%)**			
≤1ULN	2773 (58%)	361 (46%)	2412 (60%)
>1 ULN–≤2 ULN	1034 (22%)	136 (17%)	898 (22%)
>2 ULN	619 (13%)	117 (15%)	502 (12%)
Unknown	380 (8%)	170 (22%)	210 (5%)
**M category, *n* (%)**			
M0	347 (7%)	53 (7%)	294 (7%)
M1a	303 (6%)	28 (4%)	275 (7%)
M1b	466 (10%)	60 (8%)	406 (10%)
M1c	3338 (69%)	488 (62%)	2850 (71%)
Unknown	352 (7%)	155 (20%)	197 (5%)
**Brain metastases, *n* (%)**			
No	3357 (70%)	460 (59%)	2897 (72%)
Yes	1307 (27%)	285 (36%)	1022 (25%)
Unknown	142 (3%)	39 (5%)	103 (3%)

ECOG = Eastern Cooperative Oncology Group; IQR = interquartile range; LDH = lactate dehydrogenase; *n* = number; SD = standard deviation; ULN = upper limit of normal.

**Table 2:** Healthcare resource use and costs of all patients.

	**All Patients**		**Patients Who Did Not Receive Systemic Therapy**		**Patients Who Received Systemic Therapy**	
	***n* = 4806**		***n* = 784**		***n* = 4022**	
**Observation period, *months***						
Mean (SD)	18.0 (16.9)		11.7 (17.0)		19.3 (16.6)	
Median (IQR)	12.1 (5.4–25.4)		3.7 (1.4–13.2)		13.5 (6.8–26.9)	
**Deceased patients, *%***	66%		81%		63%	
	**Mean resource use (SD)**	**Mean costs (SD)**	**Mean resource use (SD)**	**Mean costs (SD)**	**Mean resource use (SD)**	**Mean costs (SD)**
**Medical imaging**						
CT scan	4.4 (4.1)	€ 684 (€ 638)	1.7 (2.1)	€ 264 (€ 331)	5.0 (4.2)	€ 766 (€ 651)
MRI scan	2.1 (2.4)	€ 589 (€ 677)	0.9 (1.6)	€ 270 (€ 458)	2.3 (2.4)	€ 651 (€ 695)
PET/CT scan	0.8 (1.3)	€ 813 (€ 1444)	0.5 (1.0)	€ 546 (€ 1088)	0.8 (1.4)	€ 865 (€ 1498)
**Genetic testing**						
Gene mutation testing	1.0 (0.2)	€ 891 (€ 185)	0.8 (0.4)	€ 753 (€ 365)	1.0 (0.1)	€ 918 (€ 102)
**Hospital visits**						
Outpatient visit	19.0 (15.6)	€ 1798 (€ 1480)	7.0 (6.6)	€ 665 (€ 624)	21.3 (15.8)	€ 2019 (€ 1497)
Daycare treatment	8.7 (10.5)	€ 2489 (€ 3020)	1.2 (2.0)	€ 345 (€ 588)	10.1 (10.9)	€ 2907 (€ 3124)
**Hospital admissions**						
Inpatient hospital day	10.4 (14.0)	€ 5150 (€ 6943)	5.4 (8.6)	€ 2656 (€ 4264)	11.4 (14.6)	€ 5636 (€ 7253)
Intensive care unit day	0.2 (1.2)	€ 213 (€ 1531)	0.1 (0.9)	€ 175 (€ 1098)	0.2 (1.3)	€ 221 (€ 1602)
**Treatment**						
Surgery	0.4 (0.9)	€ 1224 (€ 2703)	0.4 (0.8)	€ 1160 (€ 2654)	0.4 (0.9)	€ 1236 (€ 2713)
Radiotherapy	0.5 (0.6)	€ 1318 (€ 1914)	0.4 (0.5)	€ 1068 (€ 1590)	0.5 (0.7)	€ 1367 (€ 1968)
Hyperthermia	<0.1 (0.1)	€ 70 (€ 871)	<0.1 (0.1)	€ 83 (€ 949)	<0.1 (0.1)	€ 68 (€ 855)
RFA	<0.1 (<0.1)	€ 2 (€ 61)	<0.1 (<0.1)	€ 2 (€ 53)	<0.1 (<0.1)	€ 3 (€ 62)
Systemic therapy	1.4 (1.2)	€ 73,998 (€ 80,716)	NA	NA	1.7 (1.1)	€ 88,422 (€ 80,682)
**Total costs**						
Mean (SD)		€ 89,240 (€ 86,489)		€ 7988 (€ 7490)		€ 105,078 (€ 85,963)
Median (IQR)		€ 67,882 (€ 22,004–€ 126,953)		€ 5310 (€ 2800–€ 11,131)		€ 83,092 (€ 43,715–€ 141,326)
**Monthly costs**						
Mean (SD)		€ 6809 (€ 5783)		€ 2483 (€ 3191)		€ 7652 (€ 5798)
Median (IQR)		€ 5692 (€ 2584–€ 9443)		€ 1304 (€ 393–€ 3243)		€ 6526 (€ 3484–€ 10,348)

CT = computed tomography; IQR = interquartile range; MRI = magnetic resonance imaging; *n* = number; NA = not applicable; PET = positron emission tomography; RFA = radiofrequency ablation; SD = standard deviation.

**Table 3:** Healthcare costs of patients who did not receive systemic therapy stratified by vital status.

	**Deceased Patients**	**Patients Alive**
	***n* = 634**	***n* = 150**
**Observation period, *months***		
Mean (SD)	5.4 (7.9)	38.2 (19.6)
Median (IQR)	2.6 (1.1–6.3)	37.4 (19.6–58.8)
	**Mean (SD)**	**Mean (SD)**
**Medical imaging**	€ 780 (€ 772)	€ 2350 (€ 2467)
**Genetic testing**	€ 778 (€ 343)	€ 644 (€ 430)
**Hospital visits**	€ 797 (€ 881)	€ 1911 (€ 1076)
**Hospital admissions**	€ 2961 (€ 4671)	€ 2279 (€ 3827)
**Treatment**		
Surgery	€ 716 (€ 2131)	€ 3039 (€ 3652)
Radiotherapy	€ 1083 (€ 1541)	€ 1003 (€ 1789)
Hyperthermia	€ 103 (€ 1054)	€ 0 (€ 0)
RFA	€ 0 (€ 0)	€ 10 (€ 122)
**Total costs**		
Mean (SD)	€ 7219 (€ 6979)	€ 11,237 (€ 8647)
Median (IQR)	€ 4720 (€ 2474–€ 9497)	€ 9262 (€ 4425–€ 15,699)
**Monthly costs**		
Mean (SD)	€ 2981 (€ 3357)	€ 378 (€ 345)
Median (IQR)	€ 1769 (€ 765–€ 4130)	€ 293 (€ 139–€ 514)

IQR = interquartile range; *n* = number; RFA = radiofrequency ablation; SD = standard deviation.

**Table 4:** Episode and monthly costs stratified by line of therapy.

	**First Line of Therapy**	**Second Line of Therapy**	**Third Line of Therapy**
	***n* = 4022**	***n* = 1915**	***n* = 838**
**Episode duration, *months***			
Mean (SD)	11.3 (12.3)	8.9 (11.1)	7.6 (9.3)
Median (IQR)	6.6 (3.5–13.7)	4.9 (2.5–9.8)	4.2 (2.5–9.3)
**Drug, *n* (%)**			
Dacarbazine	154 (4%)	33 (2%)	29 (3%)
Ipilimumab	488 (12%)	440 (23%)	86 (10%)
Nivolumab	412 (10%)	205 (11%)	64 (8%)
Pembrolizumab	830 (21%)	370 (19%)	158 (19%)
Nivolumab plus ipilimumab	368 (9%)	249 (13%)	46 (5%)
Vemurafenib	540 (13%)	64 (3%)	53 (6%)
Dabrafenib	191 (5%)	85 (4%)	40 (5%)
Dabrafenib plus trametinib	588 (15%)	286 (15%)	233 (28%)
Vemurafenib plus cobimetinib	105 (3%)	66 (3%)	50 (6%)
Other	346 (9%)	117 (6%)	79 (9%)
**Patients with a complete episode ^1^, *%***	80%	81%	80%
	**Mean (SD)**	**Mean (SD)**	**Mean (SD)**
**Medical imaging**	€ 1349 (€ 1145)	€ 941 (€ 1109)	€ 806 (€ 937)
**Genetic testing**	€ 829 (€ 288)	€ 10 (€ 94)	€ 0 (€ 0)
**Hospital visits**	€ 2789 (€ 2764)	€ 2554 (€ 2859)	€ 2179 (€ 2314)
**Hospital admissions**	€ 2993 (€ 5525)	€ 3206 (€ 5209)	€ 2805 (€ 4456)
**Treatment**			
Surgery	€ 527 (€ 1677)	€ 375 (€ 1552)	€ 316 (€ 1466)
Radiotherapy	€ 651 (€ 1269)	€ 600 (€ 1245)	€ 574 (€1207)
Hyperthermia	€ 27 (€ 542)	€ 23 (€ 497)	€ 13 (€ 376)
RFA	<€ 1 (€ 24)	€ 1 (€ 34)	€ 4 (€ 73)
Systemic therapy	€ 49,336 (€ 49,118)	€ 51,993 (€ 47,431)	€ 43,028 (€ 43,465)
**Episode costs**			
Mean (SD)	€ 58,502 (€ 51,066)	€ 59,701 (€ 49,380)	€ 49,725 (€ 45,146)
Median (IQR)	€ 48,357 (€ 22,376–€ 80,885)	€ 50,392 (€ 22,907–€ 85,434)	€ 37,771 (€ 15,370–€ 69,036)
**Monthly costs**			
Mean (SD)	€ 8231 (€ 7374)	€ 11,939 (€ 11,463)	€ 10,366 (€ 10,415)
Median (IQR)	€ 6587 (€ 3416–€ 11,019)	€ 8439 (€ 4774–€ 14,877)	€ 7716 (€ 3974–€ 13,059)

IQR = interquartile range; *n* = number; RFA = radiofrequency ablation; SD = standard deviation.^1^ These patients either died during the line of therapy or received a new systemic therapy.

**Table 5:** Unit costs.

Resource	Unit Cost
**Medical imaging**	
CT scan	€ 154.21
MRI scan	€ 285.91
PET/CT scan	€ 1069.76
**Genetic testing**	
Gene mutation testing ^1^	€ 929.25
**Hospital visits**	
Outpatient visit	€ 94.69
Daycare treatment	€ 287.19
**Hospital admissions**	
Inpatient hospital day	€ 495.30
Intensive care unit day	€ 1234.08
**Surgery**	
Excision	€ 95.65
Lymph node dissection	€ 1734.62
Metastasectomy ^2^	€ 2999.07–€ 6239.07
**Radiotherapy**	
Short course (≤6 sessions)	€ 2034.13
Standard course (>6 sessions)	€ 4840.38
**Hyperthermia**	
Hyperthermia	€ 10,877.17
**RFA**	
RFA	€ 1490.84
**Systemic therapy**	
*Dacarbazine*	
Vial 500 mg	€ 46.33
Vial 1000 mg	€ 87.15
*Temozolomide*	
Capsule 5 mg	€ 2.60
Capsule 20 mg	€ 4.80
Capsule 100 mg	€ 17.40
Capsule 140 mg	€ 24.00
Capsule 180 mg	€ 30.40
Capsule 250 mg	€ 40.20
*Ipilimumab*	
Vial 50 mg	€ 4250.00
Vial 200 mg	€ 17,000.00
*Nivolumab*	
Vial 40 mg	€ 405.03
Vial 100 mg	€ 1012.56
Vial 240 mg	€ 2430.15
*Pembrolizumab*	
Vial 50 mg	€ 1312.18
Vial 100 mg	€ 2624.37
*Vemurafenib*	
Tablet 240 mg	€ 30.70
*Dabrafenib*	
Capsule 50 mg	€ 35.53
Capsule 75 mg	€ 52.16
*Trametinib*	
Tablet 0.5 mg	€ 54.19
Tablet 2 mg	€ 203.81
*Cobimetinib*	
Tablet 20 mg	€ 86.89
*Encorafenib*	
Capsule 50 mg	€ 24.41
Capsule 75 mg	€ 36.05
*Binimetinib*	
Tablet 15 mg	€ 34.09
*Investigational drug ^3^*	€ 0.00

CT = computed tomography; mg = milligram; MRI = magnetic resonance imaging; PET = positron emission tomography; RFA = radiofrequency ablation^. 1^ BRAF, NRAS, KIT, GNAQ, and GNA11. ^2^ Ranging from € 2999.07 for soft tissue metastases to € 6239.07 for pancreatic metastases. ^3^ Costs of investigational drugs were set at zero if the drug was given in a blinded trial or if the drug was not approved for metastatic melanoma in The Netherlands at the time of this study.

## Supplementary Materials

The following are available online at https://www.mdpi.com/2072-6694/12/4/1003/s1, Table S1: Immunotherapeutic and targeted drugs approved for the treatment of metastatic melanoma since 2011, Table S2: Baseline patient and tumor characteristics of patients who did not receive systemic therapy stratified by vital status, and Table S3: Detailed results of episode costs stratified by drug.
