# Supplementary material for: Healthcare Costs of Metastatic Cutaneous Melanoma in the Era of Immunotherapeutic and Targeted Drugs
Source: Cancers (Basel). 2020 Apr 18;12(4):1003. doi: 10.3390/cancers12041003 (PMC7225943; doi:10.3390/cancers12041003)
Supplement: Supplementary file 1 [file cancers-12-01003-s001.zip › Table S1_Leeneman_Final.docx]

**Table S1.** Immunotherapeutic and targeted drugs approved for the treatment of metastatic melanoma since 2011.

| **Immunotherapeutic drugs** | **FDA approval** | **EMA approval** |
| --- | --- | --- |
| Ipilimumab | Mar 2011 | Jul 2011 |
| Nivolumab | PT: Dec 2014 | Jun 2015 |
| Pembrolizumab | PT: Sep 2014 | Jul 2015 |
|  | TN: Dec 2015 |  |
| Nivolumab plus ipilimumab | BRAF wild-type: Oct 2015 | May 2016 |
|  | Across BRAF status: Jan 2016 |  |
| **Targeted drugs** | **FDA approval** | **EMA approval** |
| Vemurafenib | Aug 2011 | Feb 2012 |
| Dabrafenib | May 2013 | Aug 2013 |
| Dabrafenib plus trametinib | Jan 2014 | Sep 2015 |
| Vemurafenib plus cobimetinib | Nov 2015 | Nov 2015 |
| Encorafenib plus binimetinib | Jun 2018 | Sep 2018 |

EMA = European Medicines Agency; FDA = U.S. Food and Drug Administration;

PT = previously-treated patients; TN = treatment-naive patients.
