# Supplementary material for: Healthcare Costs of Metastatic Cutaneous Melanoma in the Era of Immunotherapeutic and Targeted Drugs
Source: Cancers (Basel). 2020 Apr 18;12(4):1003. doi: 10.3390/cancers12041003 (PMC7225943; doi:10.3390/cancers12041003)
Supplement: Supplementary file 1 [file cancers-12-01003-s001.zip › Table S2_Leeneman_Final.docx]

**Table S2.** Baseline patient and tumor characteristics of patients who did

not receive systemic therapy stratified by vital status.

|  | **Deceased patients** | **Patients alive** |
| --- | --- | --- |
|  | ***n = 634*** | ***n = 150*** |
| Age, years |  |  |
| Mean (SD) | 71 (12) | 64 (15) |
| Median (IQR) | 73 (64-80) | 65 (54-75) |
| Gender, *n* (%) |  |  |
| Male | 371 (59%) | 76 (51%) |
| Female | 262 (41%) | 74 (49%) |
| Unknown | 1 (0%) | 0 (0%) |
| ECOG performance status, *n* (%) |  |  |
| 0 | 90 (14%) | 65 (43%) |
| 1 | 171 (27%) | 22 (15%) |
| ≥2 | 198 (31%) | 11 (7%) |
| Unknown | 175 (28%) | 52 (35%) |
| LDH level, *n* (%) |  |  |
| ≤1ULN | 271 (43%) | 90 (60%) |
| >1ULN–≤2ULN | 127 (20%) | 9 (6%) |
| >2ULN | 115 (18%) | 2 (1%) |
| Unknown | 121 (19%) | 49 (33%) |
| M category, *n* (%) |  |  |
| M0 | 41 (6%) | 12 (8%) |
| M1a | 13 (2%) | 15 (10%) |
| M1b | 30 (5%) | 30 (20%) |
| M1c | 448 (71%) | 40 (27%) |
| Unknown | 102 (16%) | 53 (35%) |
| Brain metastases, *n* (%) |  |  |
| No | 352 (56%) | 108 (72%) |
| Yes | 259 (41%) | 26 (17%) |
| Unknown | 23 (4%) | 16 (11%) |

ECOG = Eastern Cooperative Oncology Group; IQR = interquartile range; LDH = lactate

dehydrogenase; *n* = number; SD = standard deviation; ULN = upper limit of normal.
