# Supplementary material for: Healthcare Costs of Metastatic Cutaneous Melanoma in the Era of Immunotherapeutic and Targeted Drugs
Source: Cancers (Basel). 2020 Apr 18;12(4):1003. doi: 10.3390/cancers12041003 (PMC7225943; doi:10.3390/cancers12041003)
Supplement: Supplementary file 1 [file cancers-12-01003-s001.zip › Table S3_Leeneman_Final.docx]

**Table S3.** Detailed results of episode costs stratified by drug. ^1^

|  | **Dacarbazine** | **Ipilimumab** | **Nivolumab** | **Pembrolizumab** | **Nivolumab plus ipilimumab** |
| --- | --- | --- | --- | --- | --- |
|  | ***n = 228*** | ***n = 1,034*** | ***n = 675*** | ***n = 1,356*** | ***n = 680*** |
|  |  |  |  |  |  |
| Episode duration, months |  |  |  |  |  |
| Mean (SD) | 5.6 (9.3) | 9.1 (12.3) | 12.5 (12.3) | 14.0 (12.9) | 9.6 (10.8) |
| Median (IQR) | 3.4 (2.1-5.0) | 4.8 (3.0-8.8) | 8.5 (3.6-16.4) | 9.2 (3.9-20.7) | 5.4 (2.4-12.7) |
| Drug use, mean (SD) |  |  |  |  |  |
| Number of cycles | 3.7 (3.8) | 3.2 (1.1) | 13.4 (12.1) | 10.4 (8.8) | N: 7.0 (9.4); I: 2.6 (1.1) |
| Number of days | NA | NA | NA | NA | NA |
| Patients with a complete episode ^2^, % | 96% | 89% | 62% | 67% | 64% |
|  | Mean (SD) | Mean (SD) | Mean (SD) | Mean (SD) | Mean (SD) |
| Medical imaging | € 810 (€ 677) | € 1122 (€ 1139) | € 1252 (€ 1213) | € 1322 (€ 1328) | € 1035 (€ 826) |
| Genetic testing | € 583 (€ 450) | € 374 (€ 455) | € 477 (€ 457) | € 489 (€ 459) | € 436 (€ 461) |
| Hospital visits | € 1670 (€ 1519) | € 1830 (€ 959) | € 5111 (€ 4226) | € 3925 (€ 2980) | € 2828 (€ 2650) |
| Hospital admissions | € 1877 (€ 3598) | € 3549 (€ 5724) | € 2332 (€ 4679) | € 2588 (€ 5465) | € 4070 (€ 5969) |
| Treatment |  |  |  |  |  |
| Surgery | € 219 (€ 922) | € 444 (€ 1708) | € 550 (€ 1667) | € 515 (€ 1776) | € 512 (€ 1580) |
| Radiotherapy | € 753 (€ 1277) | € 772 (€ 1336) | € 607 (€ 1247) | € 692 (€ 1346) | € 629 (€ 1251) |
| Hyperthermia | € 0 (€ 0) | € 42 (€ 676) | € 48 (€ 724) | € 8 (€ 295) | € 16 (€ 417) |
| RFA | € 0 (€ 0) | € 1 (€ 46) | € 1 (€ 29) | € 1 (€ 40) | € 0 (€ 0) |
| Systemic therapy | € 652 (€ 749) | € 70,976 (€ 28,149) | € 38,637 (€ 33,968) | € 49,485 (€ 43,072) | € 70,149 (€ 42,074) |
| Episode costs |  |  |  |  |  |
| Mean (SD) | € 6564 (€ 5090) | € 79,110 (€ 29,113) | € 49,016 (€ 38,537) | € 59,025 (€ 46,413) | € 79,675 (€ 44,196) |
| Median (IQR) | € 5142 (€ 3323-€ 8465) | € 82,536 (€ 59,172-€ 100,209) | € 38,647 (€ 19,725-€ 71,366) | € 47,315 (€ 24,658-€ 75,710) | € 72,338 (€ 47,719-€ 103,312) |
| Monthly costs |  |  |  |  |  |
| Mean (SD) | € 2027 (€ 1871) | € 17,252 (€ 11,146) | € 5,732 (€ 5,279) | € 5798 (€ 3846) | € 16,976 (€ 13,279) |
| Median (IQR) | € 1478 (€ 970-€ 2448) | € 15,920 (€ 9218-€ 23,314) | € 4,963 (€ 3148-€ 6,742) | € 5551 (€ 3577-€ 7464) | € 14,077 (€ 7036-€ 23,425) |

**Table S3.** *Continued*.

|  | **Vemurafenib** | **Dabrafenib** | **Dabrafenib plus trametinib** | **Vemurafenib plus cobimetinib** |  |
| --- | --- | --- | --- | --- | --- |
|  | ***n = 634*** | ***n = 316*** | ***n = 1,047*** | ***n = 242*** |  |
|  |  |  |  |  |  |
| Episode duration, months |  |  |  |  |  |
| Mean (SD) | 7.6 (10.4) | 6.3 (8.9) | 8.3 (8.7) | 6.4 (6.8) |  |
| Median (IQR) | 4.6 (2.6-8.0) | 3.9 (2.6-6.5) | 5.5 (3.3-9.8) | 4.4 (1.9-7.9) |  |
| Drug use, mean (SD) |  |  |  |  |  |
| Number of cycles | NA | NA | NA | NA |  |
| Number of days | 153.8 (205.5) | 120.9 (106.9) | D: 177.5 (157.2); T: 167.0 (151.5) | V: 125.7 (136.8); C: 115.2 (124.8) |  |
| Patients with a complete episode ^2^, % | 97% | 97% | 82% | 93% |  |
|  | Mean (SD) | Mean (SD) | Mean (SD) | Mean (SD) |  |
| Medical imaging | € 994 (€ 777) | € 1018 (€ 893) | € 1069 (€ 880) | € 1028 (€ 1269) |  |
| Genetic testing | € 731 (€ 367) | € 492 (€ 456) | € 434 (€ 435) | € 365 (€ 441) |  |
| Hospital visits | € 1298 (€ 1202) | € 1002 (€ 703) | € 1606 (€ 850) | € 1682 (€ 936) |  |
| Hospital admissions | € 3050 (€ 4750) | € 2725 (€ 4789) | € 3346 (€ 5051) | € 3748 (€ 6094) |  |
| Treatment |  |  |  |  |  |
| Surgery | € 262 (€ 1118) | € 387 (€ 1257) | € 468 (€ 1551) | € 377 (€ 1594) |  |
| Radiotherapy | € 719 (€ 1205) | € 611 (€ 1155) | € 509 (€ 1112) | € 376 (€ 976) |  |
| Hyperthermia | € 51 (€ 747) | € 0 (€ 0) | € 21 (€ 475) | € 0 (€ 0) |  |
| RFA | € 0 (€ 0) | € 0 (€ 0) | <€ 1 (€ 15) | € 0 (€ 0) |  |
| Systemic therapy | € 35,750 (€ 47,845) | € 24,177 (€ 21,989) | € 69,601 (€ 62,121) | € 57,344 (€ 63,435) |  |
| Episode costs |  |  |  |  |  |
| Mean (SD) | € 42,856 (€ 49,334) | € 30,411 (€ 23,569) | € 77,053 (€ 63,451) | € 64,922 (€ 65,120) |  |
| Median (IQR) | € 31,210 (€ 16,399-€ 49,791) | € 25,941 (€ 16,621-€ 37,153) | € 64,645 (€ 39,948-€ 86,178) | € 47,492 (€ 16,305-€ 83,183) |  |
| Monthly costs |  |  |  |  |  |
| Mean (SD) | € 6710 (€ 2843) | € 6460 (€ 2869) | € 12,015 (€ 7732) | € 11,947 (€ 6538) |  |
| Median (IQR) | € 6936 (€ 5113-€ 8023) | € 6590 (€ 4881-€ 7525) | € 12,146 (€ 9238-€ 13,554) | € 12,325 (€ 8369-€ 15,078) |  |

IQR = interquartile range; *n* = number; NA = not applicable; RFA = radiofrequency ablation; SD = standard deviation.

^1^ Due to low numbers of patients, costs were not separately reported for temozolomide and encorafenib plus binimetinib.

^2^ These patients either died during the line of therapy or received a new line of therapy.
